# Forensic Analysis for Source Camera Identification from EXIF Metadata

**DOI:** 10.3390/jimaging12030110

**Published:** 2026-03-04

**Authors:** Pengpeng Yang, Chen Zhou, Daniele Baracchi, Dasara Shullani, Yaobin Zou, Alessandro Piva

**Affiliations:** 1 Hubei Key Laboratory of Intelligent Vision Based Monitoring for Hydroelectric Engineering, China Three Gorges University, Yichang 443002, China; ppyang@ctgu.edu.cn (P.Y.); zc@ctgu.edu.cn (C.Z.); 2 College of Computer and Information Technology, China Three Gorges University, Yichang 443002, China; 3 Department of Information Engineering, University of Florence, Via di S. Marta 3, 50139 Firenze, Italy; daniele.baracchi@unifi.it (D.B.); dasara.shullani@unifi.it (D.S.);

**Keywords:** source camera identification, multimedia forensics, EXIF metadata, type-aware word embedding, anomalous behaviors

## Abstract

Source camera identification on smartphones constitutes a fundamental task in multimedia forensics, providing essential support for applications such as image copyright protection, illegal content tracking, and digital evidence verification. Numerous techniques have been developed for this task over the past decades. Among existing approaches, Photo-Response Non-Uniformity (PRNU) has been widely recognized as a reliable device-specific fingerprint and has demonstrated remarkable performance in real-world applications. Nevertheless, the rapid advancement of computational photography technologies has introduced significant challenges: modern devices often exhibit anomalous behaviors under PRNU-based analysis. For instance, images captured by different devices may exhibit unexpected correlations, while images captured by the same device can vary substantially in their PRNU patterns. Current approaches are incapable of automatically exploring the underlying causes of these anomalous behaviors. To address this limitation, we propose a simple yet effective forensic analysis framework leveraging Exchangeable Image File Format (EXIF) metadata. Specifically, we represent EXIF metadata as type-aware word embeddings to preserve contextual information across tags. This design enables visual interpretation of the model’s decision-making process and provides complementary insights for identifying the anomalous behaviors observed in modern devices. Extensive experiments conducted on three public benchmark datasets demonstrate that the proposed method not only achieves state-of-the-art performance for source camera identification but also provides valuable insights into anomalous device behaviors.

## 1. Introduction

From social media sharing to news reporting, and from e-commerce to judicial evidence, digital images have become an indispensable medium for information transmission. According to statistics [1,2], the amount of such digital media generated and transmitted each year globally is growing exponentially, and this trend is expected to continue in the coming years. However, the widespread availability of image editing tools and the evolution of tampering techniques, such as Deepfakes [3], have posed unprecedented challenges to the reliability and authenticity of images. In this context, multimedia forensics has become crucial for verifying the origin of images, combating misinformation, and addressing digital crimes [4,5]. Especially on social media, where issues such as misinformation and illegal content are more elusive, recent efforts to address these types of issues, such as profile linking, should be taken into consideration [6,7,8].

Source camera identification is a core task in multimedia forensics, aiming to accurately trace and confirm the physical device that captured an image by analyzing the device features embedded in the image. Currently, the most well-known and reliable technique for source camera identification is based on Photo-Response Non-Uniformity (PRNU), which originates from the inhomogeneity of silicon wafers and imperfections introduced during the sensor manufacturing process. However, its performance has been seriously affected by the rapid development of computational photography technologies. Iuliani et al. [9] conducted an extensive testing campaign on more than 33,000 Flickr images captured by 45 smartphones and 25 DSLR camera models released in recent years. Their results revealed that many brands, including Samsung, Huawei, Canon, Nikon, Fujifilm, Sigma, and Leica, present a severe risk of correlated patterns appearing in images from different devices, leading to a substantial increase in the false positive rate of PRNU-based detection. Du et al. [10] further constructed a large-scale benchmark dataset, ForensiCam-215K, comprising images captured by 130 modern smartphones, and evaluated the performance of PRNU-based methods. They found that images captured by recent smartphones exhibit significantly degraded performance and considerable intra-device variation in their PRNU patterns.

Although the anomalous behaviors of PRNU-based methods on modern imaging devices have attracted considerable attention in the image forensics community, most existing studies [11,12,13] have primarily focused on mitigating individual factors that may induce such abnormalities, such as high dynamic range imaging, portrait mode processing and lens radial distortion. These approaches are typically based on the assumption that such factors are the root causes of anomalous behaviors, leading to the design of corresponding correction schemes that are subsequently validated on well-constructed datasets. However, these methods lack the capability to uncover new underlying causes of anomalous behaviors when applied to more complex datasets containing diverse types of images. Therefore, there is an urgent need to develop an effective and automated analytical framework capable of systematically identifying and explaining such abnormalities.

To address this challenge, we propose an explainable and fully automated forensic framework based on EXIF metadata to investigate the underlying causes of the anomalous behaviors observed in PRNU-based methods. Specifically, we design a type-aware word embedding scheme to construct feature representations of EXIF metadata. The EXIF tags are automatically categorized into numerical and textual types via computational rules, eliminating the need for manual intervention. This approach preserves the semantic relationships among metadata attributes. A decision tree model is then employed for final reasoning, providing interpretable visual explanations of the decision process. Extensive experiments conducted on three widely used benchmark datasets demonstrate the effectiveness and interpretability of the proposed framework.

It is important to explicitly define the operational scope and assumptions of this study. Since the primary objective is to resolve the source identification dilemmas (specifically the unexpected False Positive and False Negative results) induced by intrinsic computational photography mechanisms, our analysis focuses on diagnosing internal device behaviors rather than detecting external adversarial attacks. Consequently, this framework operates under the assumption that the EXIF metadata is complete and authentic, serving as a reliable basis for interpreting device-specific imaging algorithms. This study prioritizes the effective utilization of available metadata to establish a precise diagnostic link between capture modes and hardware fingerprints in reliable forensic settings.

More concretely, the main contributions of this paper are as follows:

- We propose a type-aware word encoding scheme that systematically categorizes EXIF information into numerical and textual types, representing each type separately in order to better capture its discriminative information.
- By employing an interpretable machine learning model and visualizing its decision structure, the proposed method not only enables efficient device identification but also provides a transparent trace of the entire classification process, offering intuitive explanations for forensic conclusions.
- We demonstrate the practical forensic value of the proposed framework by achieving state-of-the-art performance on three benchmark datasets. More significantly, through interpretable case studies, we uncover novel insights into the anomalous behaviors of modern imaging devices, moving beyond mere classification accuracy to provide actionable forensic explanations.

The rest of the paper is organized as follows. The next section reviews related work on source camera identification. Section 3 provides a detailed explanation of the proposed method. Section 4 validates and analyzes the performance of the method through carefully designed experiments. Finally, in Section 5, the analyses are presented, and the conclusions are drawn.

## 2. Related Work

Source camera identification constitutes a fundamental research topic in digital image forensics, aiming to accurately determine the source device of an image. Early studies primarily relied on the analysis of device-specific physical characteristics embedded in images, such as lens distortion [14], color filter array (CFA) interpolation artifacts [15] and photo-response non-uniformity (PRNU) patterns [16]. In recent years, deep learning-based techniques [17,18,19,20] have also been extensively explored for source camera identification and have achieved remarkable performance. It should be noted that, compared with both physical feature-based and deep learning-based approaches, PRNU-based methods have demonstrated superior reliability and robustness, enabling the identification of individual imaging devices even within the same camera model.

However, the rapid advancement of computational photography technologies has introduced significant challenges to the reliability of PRNU-based methods. The complex imaging pipelines employed in modern smartphones can severely distort or suppress PRNU signals, thereby hindering their accurate extraction and reliable matching [9,10]. Specifically, these challenges manifest in two major aspects: (1) an increased probability of false negatives [13,21,22], where images captured by the same device exhibit reduced correlation, and (2) an increased probability of false positives [23,24,25], where images captured by different devices show unexpectedly high correlation with the query device. These issues have drawn considerable attention from the digital forensics community, motivating extensive research to investigate their underlying causes and potential mitigation strategies.

Regarding the issue of false negatives, existing studies have primarily focused on three influencing factors: ISO settings, lens radial distortion, and high dynamic range (HDR) imaging. Quan et al. [22] verified that an increase in ISO sensitivity amplifies electronic noise, which obscures the distinctiveness of the PRNU pattern and significantly degrades inter-device fingerprint discriminability. Montibeller et al. [13] demonstrated that radial distortion correction applied to wide-angle lenses often involves non-linear geometric transformations, resulting in global or local misalignment of the PRNU pattern and thereby impeding accurate fingerprint matching. Darvish et al. [21] further pointed out that HDR images are produced through multi-exposure fusion, where the alignment process requires local translation adjustments across frames. This operation introduces pixel-level misalignments of the PRNU signal in localized regions of the image, substantially reducing the Peak Correlation Energy (PCE) during matching.

For the issue of false positives, the common cause can be attributed to Non-Unique Artifacts (NUAs). Iuliani et al. [9] were among the first to report that devices ranging from modern smartphones (e.g., Huawei, Samsung) to professional cameras (e.g., Fujifilm, Sony) exhibited notable false positive rates. They further noted that NUAs leading to false matches are primarily associated with unusual shooting modes, brand-specific proprietary technologies, and model-dependent in-camera processing. Butora et al. [24,26] highlighted that general-purpose software such as Adobe Lightroom and Camera Raw embed a periodic pattern of 128 × 128 pixels. This pattern, as a device-independent NUA, exhibits a strong correlation with the PRNU signal, resulting in systematic false matches between images originating from different devices but processed using the same software. Furthermore, several studies [12,23,27] reported that synthetic defocus processing in portrait mode, designed to achieve background blur, introduces artifacts that are shared across devices. More recently, Vázquez et al. [25] discovered that Samsung Galaxy S/A series smartphones embed a diagonal periodic pattern in their default imaging mode, which remains highly consistent across devices and leads to false positive rates exceeding 95%.

Even though some studies have addressed the aforementioned anomalous behaviors, we emphasize that automatically identifying the underlying causes of false negatives and false positives remains highly challenging. As reported by Tamiazzo et al. [28], even a detailed analysis in both the pixel and frequency domains failed to provide conclusive evidence explaining the origin of false positives. Fortunately, the Exchangeable Image File Format (EXIF), which contains crucial metadata embedded in images, can offer valuable insights for this task. As an example, Iuliani et al. [9] examined the EXIF metadata and observed that most false positive samples exhibited the tag Custom Rendered: Portrait HDR, indicating that these images were captured in portrait mode. This finding suggests that EXIF metadata analysis can greatly enhance the understanding of the anomalous behaviors exhibited by PRNU-based methods on modern imaging devices. However, current automated approaches for EXIF metadata analysis are still lacking, as existing techniques primarily rely on one-to-one manual comparison. Although preliminary EXIF-based schemes have been proposed for source camera identification, they are not directly applicable to addressing this problem. Hence, there is an urgent need to develop an automated EXIF metadata analysis method.

## 3. Proposed Method

**Figure 1:**
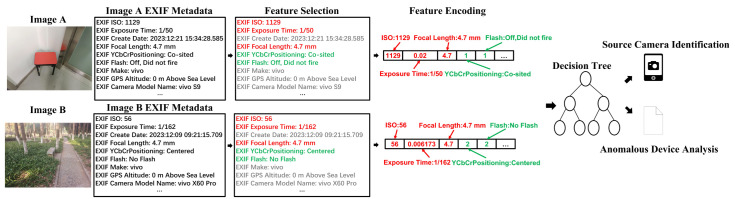
The overall architecture of our methodology. The red-highlighted content corresponds to the encoding of numerical metadata, the green-highlighted content represents the encoding of textual metadata, and the gray-highlighted content denotes the irrelevant metadata excluded during feature selection.

EXIF metadata in digital images is essentially a collection of field-value pairs, and its unstructured format makes it difficult for machine learning models to process directly. Therefore, we present a new framework that constructs a type-aware metadata feature representation scheme, followed by the development of an interpretable classification model, as shown in Figure 1. It begins with a feature selection phase to exclude irrelevant feature information. Subsequently, we conduct a thorough analysis of the data types in the remaining metadata and design a type-aware feature encoding scheme. Specifically, for numerical metadata, we directly use its value as a feature. For textual data, we apply ordinal label encoding to transform it into a discrete numerical sequence. Finally, the encoded feature vectors are fed into an interpretable classification model for source camera identification and the interpretability analysis of anomalous devices.

### 3.1. Feature Extraction and Selection

To comprehensively obtain image metadata, this study employs the professional metadata analysis tool ExifTool-12.96 [29] for an in-depth analysis of each image. This tool is capable of extracting the most complete metadata records from image files, covering standard EXIF data as well as manufacturer-specific tags. The extracted information spans a wide range of details, from capture parameters to file attributes and other related dimensions. For notational convenience, the following symbols are defined. $X_{i}=\left\{x_{1},x_{2},x_{3}\ldots x_{j}\ldots x_{n}\right\}$ and $C_{i}\in \left\{1,2,3\ldots s\right\}$ denote the feature representation and the ground-truth label of the *i*-th image, respectively, where *n* is the number of features, *j* denotes the index of the features, and *s* is the number of classes. Furthermore, let $w_{j}^{i}$ and $v_{j}^{i}$ represent the field and corresponding value of the *j*th EXIF metadata item for the *i*th image, respectively.

To ensure that the model learns intrinsic features governed by internal imaging parameters rather than relying on semantic cues directly associated with device identity, we first employ a feature selection strategy grounded in prior knowledge of the imaging process to eliminate irrelevant EXIF metadata. In general, the excluded metadata can be categorized into three groups. First, capture time and geolocation information are strictly removed, as they are context-dependent and lack consistent discriminative characteristics across devices of the same category. For the remaining retained tags, their types are automatically identified via computational rules without manual intervention. Second, device identifiers such as manufacturer, model, and software version are excluded because they explicitly encode device identity rather than contributing to the inference of intrinsic imaging patterns. Finally, parameters related to thumbnail and original image size are discarded, since these attributes are typically determined by post-processing software rather than originating from the camera’s physical imaging process. Examples of the excluded metadata are summarized in Table 1.

After the feature selection process, the final feature set included in the analysis covers multiple dimensions closely related to the camera’s inherent imaging characteristics. As shown in Table 2, these features are primarily categorized into three types: First, exposure parameters reflecting core imaging conditions, such as aperture, shutter speed, and ISO. Second, optical parameters indicating the physical state of the lens, such as focal length and focus mode. Finally, internal camera processing parameters, such as white balance, metering mode, and scene type. We formulate the feature selection process as follows:

(1) $$w_{j}^{i}=\left\{\begin{array}{cc} ⌀, & \mathrm{if}\;w_{j}^{i}\;\mathrm{belongs}\;\mathrm{to}\;\mathrm{irrelevant}\;\mathrm{metadata}\\ w_{j}^{i}, & \mathrm{otherwise} \end{array}\right.$$

### 3.2. Type-Aware Feature Encoding

After the feature selection process, we obtained a subset of metadata that reflects the device’s imaging configuration and intrinsic processing characteristics. Next, inspired by text-to-vector techniques in natural language processing, we design a word embedding scheme to encode metadata information. Specifically, a type-aware feature encoding method is proposed that preserves critical information according to the inherent data types. Based on public EXIF metadata documentation, EXIF fields are categorized into two types: numerical scalars (e.g., ISO, focal length) and categorical text (e.g., scene mode, metering mode). For numerical features, continuity and relative relationships are preserved. Categorical text features are encoded using ordinal label encoding to maintain their discrete structure.

#### 3.2.1. Numerical Features Encoding

Numerical EXIF metadata, generated and recorded during the camera imaging process, holds clear physical meaning and contains rich information regarding the specific imaging parameters and device settings employed during capture, such as exposure values and optical configurations. Directly using these numerical values is the optimal strategy to preserve their original information content and physical interpretability. The encoding process for numerical metadata in this method is highlighted in red in Figure 1.

For inherently numerical metadata (e.g., ISO: 56), the value is directly used as the encoding embedding. When the metadata value is represented as a string containing fractions or mathematical expressions (e.g., shutter speed: “1/162”), the corresponding numerical result is calculated and encoded. Similarly, for values with unit symbols (e.g., focal length: “4.7 mm”), the numerical part is extracted, and all non-numeric characters are removed. Furthermore, to ensure the completeness of the feature set, any missing metadata fields in the image are prefilled with a zero value. This approach effectively addresses the heterogeneity of the original data and provides a reliable foundation for subsequent model training.

#### 3.2.2. Textual Features Encoding

Unlike numerical features, textual metadata consists of a series of discrete text labels. To effectively integrate such non-numeric information into a unified framework, we adopt an ordinal label encoding scheme that establishes a deterministic mapping from each unique text value in the feature domain to positive integers. Specifically, when a new unique text label is encountered for the first time, it is assigned an incrementing integer identifier starting from 1. At the same time, all entries identified as missing or unknown are uniformly mapped to the integer 0, systematically addressing the issue of missing data. The part highlighted in green in Figure 1 illustrates an example of the “YCbCrPositioning” metadata encoding for two images. In this example, the value “Co-sited” in image A is encoded as the integer 1, whereas the value “Centered” in image B is encoded as the integer 2.

There are two main advantages associated with type-aware feature encoding: (1) it ensures lossless information conversion while maintaining high computational efficiency, and (2) it directly maps each category to a unique integer, generating compact, low-dimensional numerical vectors, which effectively avoids the feature space explosion and data sparsity issues that may arise with methods like one-hot encoding. Therefore, a type-aware feature encoding strategy aligns closely with the goal of constructing an efficient and interpretable feature space. The above feature encoding process can be formulated as follows:

(2) $$x_{j}=\left\{\begin{array}{cc} v_{j}^{i}, & w_{j}^{i}\;\mathrm{is}\;\mathrm{of}\;\mathrm{purely}\;\mathrm{numeric}\;\mathrm{type}\\ p(v_{j}^{i}), & w_{j}^{i}\;\mathrm{is}\;\mathrm{of}\;\mathrm{textual}\;\mathrm{numeric}\;\mathrm{type}\\ o(v_{j}^{i}), & w_{j}^{i}\;\mathrm{is}\;\mathrm{of}\;\mathrm{textual}\;\mathrm{type} \end{array}\right.$$

where p() denotes the result of a mathematical expression, and o() represents the order label encoding.

### 3.3. Feature Classification

After constructing the feature vectors X, an appropriate classification model needs to be selected to balance classification performance with the interpretability of its output. The choice of the model is primarily based on three considerations. First, the model must achieve competitive classification accuracy to ensure reliable identification. Second, the model must have high inference efficiency to meet the demand for rapid processing of large-scale image data in practical forensic scenarios. Third, and most critically for forensic applications, the model should provide intuitive and understandable decision-making criteria to support subsequent result analysis. Based on these requirements, we employ a decision tree model [30] as the classifier. This model has demonstrated good performance in video container forensics [31], and its effectiveness and applicability provide further methodological support for this study.

This study employs the Classification and Regression Trees (CART) algorithm [32], which follows the standard “grow and prune” procedure. During the growing phase, the algorithm uses a forward selection strategy to recursively perform binary splits starting from the root node. At each internal node, the algorithm evaluates all features and their potential split points, selecting the optimal split that maximizes the reduction in impurity of the child nodes. The algorithm chooses the split that results in the largest reduction in the weighted sum of impurities for the child nodes. This recursive partitioning process continues until a preset stopping criterion is met (e.g., reaching a minimum node size or achieving perfect purity), ultimately resulting in a “maximal tree” that may overfit the training data.

To address the overfitting issue, a pruning phase follows. During this phase, a penalty term is introduced to balance the model’s fit to the training data and its structural complexity. Specifically, the process starts from the “maximal tree” and generates a sequence of nested subtrees with decreasing complexity through stepwise pruning. The final model is selected from this sequence based on the evaluation results from an independent validation set, choosing the subtree with the best predictive performance.

## 4. Experiments and Evaluation

To evaluate the effectiveness of the proposed method, we conducted experiments on three large-scale benchmark datasets. First, we evaluated the proposed method on source camera identification, considering both brand and model levels to assess its generalizability and discriminative granularity. Additionally, to gain a deeper understanding of the anomalous behaviors of PRNU-based methods, we performed an attribution analysis of the false negatives and false positives.

### 4.1. Dataset

Three datasets are used for evaluation, including two large-scale public datasets, VISION [33] and ForensiCam-215K [10], as well as one dataset constructed by us.

VISION [33] is one of the most widely used public datasets, comprising 34,427 images and 1914 videos from 35 smartphone models across 11 major brands. The images include both original versions and versions compressed by social platforms such as Facebook, YouTube, and WhatsApp. It is important to note that these platforms often remove EXIF metadata during the upload process, which poses a significant challenge for metadata-based forensics [34]. Since the primary challenge for metadata-based forensics on social media is the stripping of EXIF tags rather than compression artifacts, this study exclusively utilizes the original images to ensure metadata availability, totaling 11,732.

ForensiCam-215K [10] is a recently constructed large-scale image forensics dataset with extensive device coverage and greater diversity in shooting modes. It contains over 215,000 media files from 130 modern smartphones across 10 leading brands, including 204,444 images and 10,652 videos. The comprehensiveness of this dataset is reflected not only in the number of devices and brand coverage but also in its systematic inclusion of three major camera types (main, wide-angle, telephoto) and six shooting modes (default, night, HDR, high-resolution, AI, and filter). This design enables effective evaluation of the proposed method’s stability under different imaging conditions.

A new dataset, HDRPlus [35], was collected by our team, comprising images from 28 smartphone devices across four brands and covering both standard dynamic range (SDR) and high dynamic range (HDR) images. To simplify the analysis, only the SDR images from this dataset were used for subsequent experiments.

### 4.2. Experimental Setup

To comprehensively evaluate the performance of the proposed method and assess its stability under different data splits, the entire process, from data partitioning to model evaluation, is independently repeated three times to obtain robust and reliable performance estimates. In each experiment, the data are randomly split into training and test sets at a 50%:50% ratio. The reported results are the averages over the three repetitions.

Accuracy, a commonly used metric in classification tasks, is adopted as the primary evaluation criterion. It provides an intuitive measure of the overall proportion of correctly classified instances. The proposed decision tree model is formulated to handle the source identification task as a multi-class classification problem directly, distinguishing among all device categories simultaneously. The definition of accuracy is given as follows:

(3) $$ACC=\frac{N_{correct}}{N_{total}}$$

where $N_{correct}$ represents the number of correctly classified instances, and $N_{total}$ is the total number of test samples. Since the sample sizes of each category in the utilized datasets are approximately balanced, accuracy serves as a comprehensive and reliable measure of the model’s overall classification performance.

### 4.3. Experimental Results

#### 4.3.1. Source Camera Identification: Brand-Level and Model-Level Analysis

The most representative work in current EXIF metadata-based source camera identification is the equivalence class method [36,37]. The core of the equivalence class method lies in constructing an image signature. If multiple cameras share the same signature, they are grouped into the same equivalence class. The size of an equivalence class reflects the uniqueness of the signature. By comparing the signature of the test image with the known camera signature database, its equivalence class can be determined, thereby inferring the camera’s brand and model. This work [36] constructs an image signature by extracting the number of entries from the EXIF metadata. These features include the number of entries in five standard image file directories, the number of additional image file directories, the total number of entries in the additional directories, and the number of metadata parsing errors.

It is important to note that the equivalence class method aims to assess the uniqueness of device configurations across the entire database, with its core metric being the distribution of equivalence class sizes (i.e., how many device configurations are unique). In contrast, our decision tree method is a supervised learning classifier, and its performance is measured by classification accuracy. Although the evaluation dimensions differ, the goal of both methods is the same: to explore the potential of metadata for device identification.

In the brand-level identification task, as shown in Table 3, the proposed method achieved near-perfect classification performance, with an average accuracy exceeding 99%, significantly outperforming the baseline method. In the model-level identification task, the proposed method also maintained exceptionally high classification accuracy across all three datasets. In contrast, the device configuration uniqueness ratio measured by the baseline equivalence class method exhibited instability across different datasets, with a fluctuation range between 55% and 84.4%.

**Figure 2:**
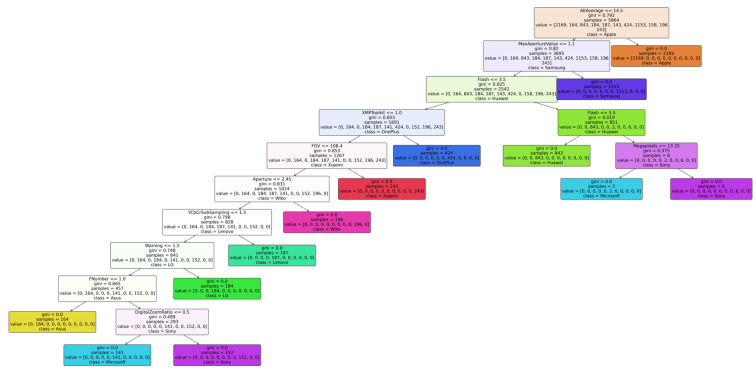
Visualization results of the brand-level decision tree on the VISION dataset.

**Figure 3:**
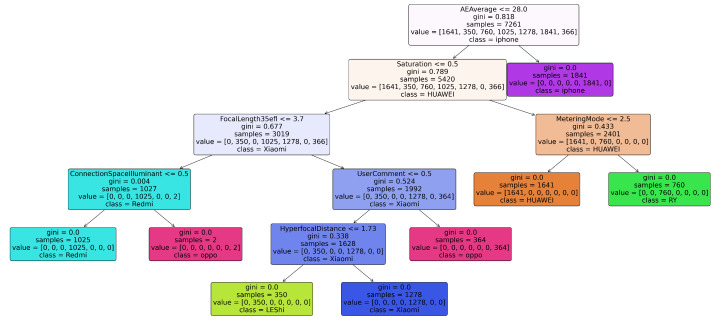
Visualization results of the brand-Level decision tree on the HDRPlus dataset.

To gain a deeper understanding of the decision-making mechanism of the proposed method in the brand identification task and verify its interpretability, this study conducted a visualization analysis of the brand classification decision trees trained on the VISION [33] and HDRPlus [35] datasets. Figure 2 and Figure 3 display the decision tree structures for the two datasets, with the results intuitively presenting the key metadata fields used by the model in the classification process, along with their corresponding decision paths.

Based on the analysis of the decision tree decision paths in the VISION [33] and HDRPlus [35] datasets, it reveals that key metadata features exhibit highly consistent discriminative power across different datasets. For instance, “AEAverage” (auto exposure average) serves as the root node feature for identifying Apple devices in both datasets, reflecting the brand’s systematic and cross-device consistency in exposure control strategies. Furthermore, different brands rely on differentiated feature combinations for effective distinction: Samsung devices are strongly correlated with “MaxApertureValue”, while OnePlus can be uniquely identified by “XMPToolkit”. Huawei and Honor devices are distinguished by the combination of “Saturation” and “MeteringMode”, while brands such as Redmi and OPPO are closely associated with parameters like “FocalLength35efl” and “UserComment”.

#### 4.3.2. Comparison with Deep Learning Baseline

To further validate the effectiveness of the proposed framework, we benchmarked our decision tree-based method against a state-of-the-art deep learning classifier. It is worth noting that deep learning approaches specifically tailored for EXIF metadata forensics are currently scarce. Therefore, to establish a robust baseline, we drew inspiration from a state-of-the-art deep learning framework designed for a structurally analogous task: file fragment classification [38]. Specifically, we employed a DL framework incorporating Byte Self-Attention (BSA) and Channel Self-Attention (CSA) modules, which is designed to capture complex contextual dependencies in raw file data. We trained and evaluated this baseline model on the VISION, HDRPlus, and ForensiCam-215K datasets under the same experimental conditions as our method.

Table 4 presents the quantitative comparison results. It can be observed that the proposed method yields performance highly comparable to, and in key aspects surpassing, the complex deep learning baseline.

These results indicate that for structured EXIF metadata, the proposed type-aware embedding scheme combined with a decision tree is sufficient to capture highly discriminative device fingerprints. Unlike deep neural networks, our framework achieves state-of-the-art accuracy, particularly on fine-grained model identification, while maintaining computational efficiency and full interpretability. This confirms that with effective feature encoding, lightweight models can outperform complex deep learning architectures in metadata forensics.

#### 4.3.3. False Negative Attribution Analysis

**Figure 4:**
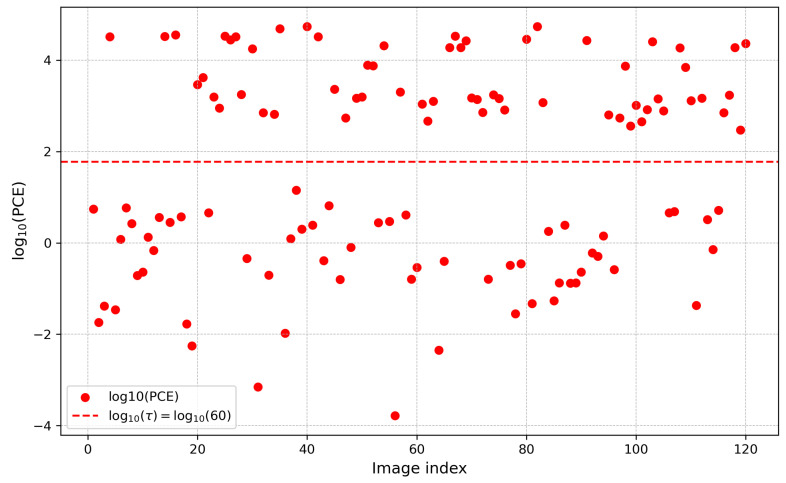
PCE distribution for Huawei Honor 6 Plus.

We analyzed devices with PRNU anomalies across three datasets and found that the issue was most prominent in the HDRPlus [35] dataset, with the Huawei Honor 6Plus as a representative example. We conducted a visual analysis of the PCE for this device, as shown in Figure 4, and observed that its value distribution was highly dispersed, with significant anomalous correlations present in some images. This revealed severe inconsistencies in the device’s internal imaging fingerprint. To investigate the cause of these anomalies, we constructed a decision tree model for the device, utilizing the model’s interpretability to identify the key metadata features responsible for the instability in PRNU.

**Figure 5:**
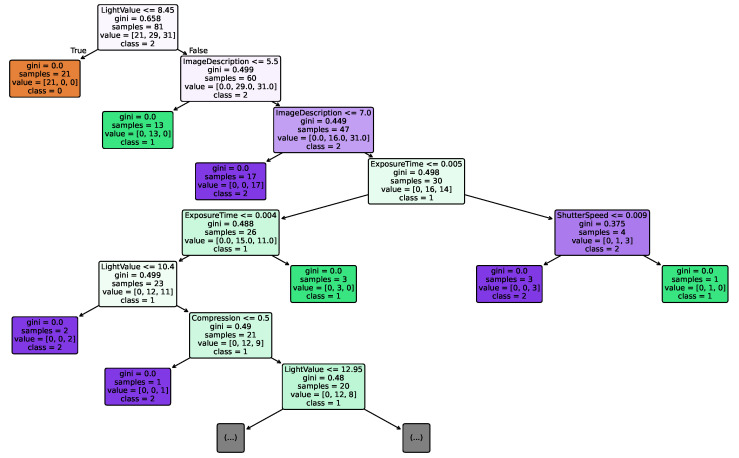
Visualization of the decision tree for Huawei Honor 6Plus (displaying the top 7 levels).

To establish an interpretable link between EXIF metadata and PRNU stability, we classified all sample images of Huawei Honor 6Plus into three categories based on the Peak Correlation Energy (PCE) ratio: FLAT images with uniform content, images with PCE values below the threshold of 60, and images with PCE values above 60. Specifically, we assigned labels 0, 1, and 2 to these three categories. FLAT images typically serve as reference frames in PRNU analysis, low PCE images represent “anomalous” images where PRNU extraction or matching failed, and high PCE images represent normal images with consistent PRNU patterns. Based on this classification framework, we employed the proposed type-aware encoding method for feature representation and trained a decision tree classifier aimed at predicting the category of each image based on its EXIF metadata, thereby revealing the intrinsic relationship between imaging metadata and PRNU stability. The decision tree model achieved an average accuracy of 85.6% on this task, validating the effectiveness of the model and suggesting a correlation between EXIF metadata and PRNU stability. Given the complexity of the full structure, Figure 5 illustrates the top 7 levels of the decision tree. This depth is sufficient to reveal the primary decision boundaries and the most discriminative metadata features, while maintaining readability.

Table 5 summarizes the analysis of key EXIF metadata affecting the PRNU stability extraction of the Huawei Honor 6Plus device. The analysis indicates that the initial separation between FLAT and NAT images is based on the “LightValue” feature. When this feature value is less than or equal to 8.45, the sample is classified as a FLAT image; otherwise, it is classified as a NAT image. In the analysis of NAT images, the “ImageDescription” serves as the key splitting node. Specifically, images with the “sdr” (Standard Dynamic Range) description correspond to low PCE value anomalous samples, while images with the “dav” (Depth of Field Aperture View) description are classified as high PCE value normal samples. Images with the “dsr” (Dual Camera Super Resolution) description contain both normal and anomalous samples, and this mixed characteristic explains the key discriminative role of this category in the decision path. In addition to “ImageDescription”, metadata such as “Compression” (compression type), “Exposure Time”, and “Shutter Speed” collectively form the complete decision process. This result confirms the systematic relationship between imaging mode configuration and PRNU extraction stability, providing interpretable evidence for understanding the anomalous behavior of PRNU in modern computational photography pipelines.

#### 4.3.4. False Positive Attribution Analysis

**Figure 6:**
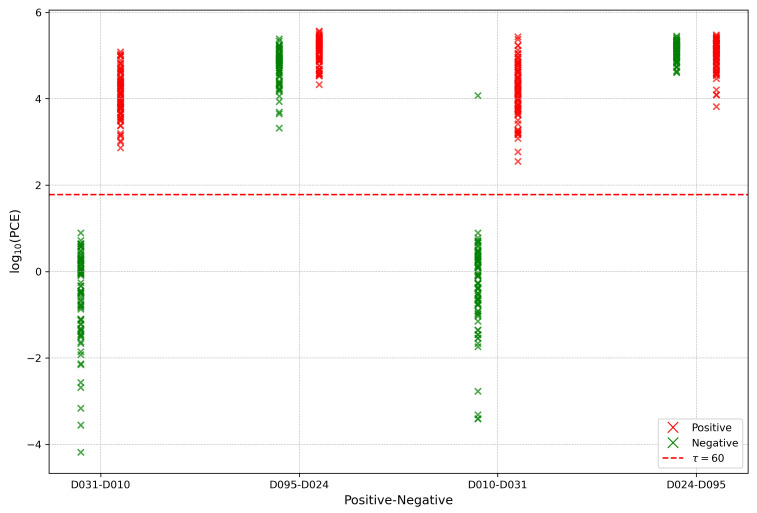
PCE distribution of anomalous and normal devices.

We next focus on analyzing the occurrence of false positives (FP) in source camera identification. Among the three datasets considered in this study, significant FP cases were observed in the ForensiCam-215K dataset. Specifically, the misclassification rates of devices D024_Vivo_X60Pro and D095_Vivo_X60 were extremely high, with their false positive rates reaching 0.96 and 1.00, respectively. We first compared these anomalous devices with normal devices from the same manufacturer (Vivo Mobile Communication Co., Ltd., Dongguan, China), namely D010_Vivo_S9 and D031_Vivo_S9, and conducted a visual analysis of the Peak to Correlation Energy (PCE) distribution across the four devices, as shown in Figure 6. The figure clearly shows that the PCE values for devices D024 and D095 are above the threshold of 60 for both negative and positive matches, indicating highly similar PRNU features that cannot be distinguished. Based on this, we conducted an attribution analysis to explore the underlying causes of this similarity.

To analyze this anomaly, we labeled the images captured by the anomalous device pair (D024 and D095) as category 1, while the images captured by the normal devices from the same brand (D010 and D031) were labeled as category 2. By converting the EXIF metadata of all images into type-aware word embedding features and feeding them into a decision tree classifier for training, we leveraged the model’s interpretability to identify the causes of the anomalies. The decision tree model achieved an average accuracy of 100% in distinguishing anomalous from normal devices, indicating its complete capability to differentiate between the two device types.

**Figure 7:**
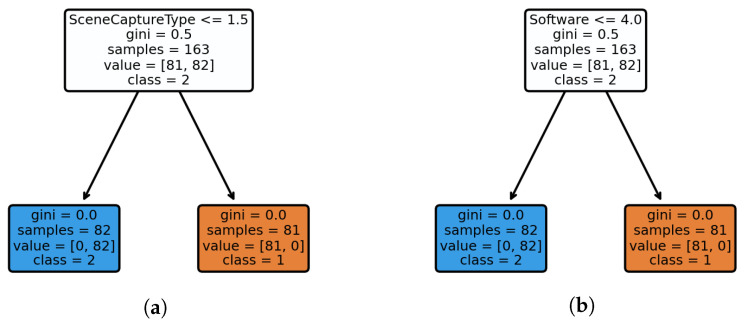
Visualization of the decision tree path for mismatched source camera identification. (**a**) Splitting feature: “SceneCaptureType”; (**b**) Splitting feature: “Software”.

As shown in Figure 7, we performed a visual analysis of the decision tree. From Figure 7a, it can be concluded that the “SceneCaptureType” metadata plays a decisive role at the root node. All images from the mismatched devices (D024 and D095) are labeled as “Portrait,” while images from the normal devices (D010 and D031) are either labeled as “Standard” or missing. Systematic validation of the dataset indicates that this anomalous value pattern is unique to D024 and D095. This finding is consistent with conclusions from existing research [12,23,27].

Furthermore, as shown in Figure 7b, at subsequent nodes, the “Software” field provides the key discriminative criterion. Specifically, the “Software” field for all mismatched devices is uniformly labeled as “SAMSUNG,” while normal devices are labeled as “MediaTek Camera Application” or have the field missing. These results demonstrate that the proposed framework is effective in exploring the underlying causes of anomalous behaviors. We further investigated this observation and found that David et al. [25] reported similar findings on Samsung devices. They claimed that certain Samsung models introduce a diagonal periodic pattern in the captured images, resulting in strong correlations across different devices. This suggests that the two Vivo devices analyzed in our study may have adopted Samsung-related technologies for image acquisition. Additionally, we conducted a preliminary frequency-domain analysis on images from the D024 and D095 devices and observed periodic signal characteristics consistent with the aforementioned description.

The study demonstrates that the proposed EXIF metadata analysis framework not only diagnoses anomalies within devices but is also applicable to explaining mismatched behavior between devices. By leveraging high-level semantic information such as “SceneCaptureType” and “Software”, this method transforms the elusive pixel-domain correlation issues into interpretable imaging context differences. This further highlights the complementary value and immense potential of EXIF metadata in building more reliable and interpretable source camera identification systems.

## 5. Conclusions

In the face of the significant challenges posed by computational photography technologies to traditional PRNU methods, we propose a novel forensic analysis framework. The core of this framework lies in transforming EXIF metadata into type-aware word embeddings and utilizing a decision tree model to achieve high-accuracy device identification and in-depth behavioral analysis.

We first confirmed the outstanding performance of the proposed method in brand and model-level identification tasks. By deeply leveraging the specific content of EXIF metadata, rather than relying solely on its structural information, this method achieved state-of-the-art performance across three public datasets, particularly achieving near-perfect accuracy in brand classification on the VISION and HDRPlus datasets. More importantly, through the visualization analysis of these high-precision decision tree models, we clearly revealed the decision-making logic behind the model’s perfect classification.

In addition, the method successfully linked the internal imaging instability of devices to specific capture modes recorded in the metadata (such as “ImageDescription”) by conducting in-depth studies of individual cases with anomalous PRNU performance. We also systematically uncovered and explained the root cause of mismatches between devices. Through comparative experiments and analysis, it was found that mismatched device pairs exhibit a high degree of consistency at the metadata level (e.g., “SceneCaptureType” and “Software”), indicating that the root cause of mismatches lies in shared computational photography algorithms between devices. These algorithms dominate the entire imaging process, and their post-processing operations overwrite the device’s unique hardware fingerprint (PRNU) while embedding shared artifacts, which are non-unique to the devices and inherent to the algorithm itself.

We also acknowledge that the proposed method still presents several limitations. First, its effectiveness is highly dependent on the completeness and authenticity of the metadata. When the metadata is manipulated or partially missing, the performance, particularly in source camera identification, may decline. Nevertheless, the method demonstrates strong capability in detecting anomalous camera behavior when the images are collected under well-controlled conditions, which represents the primary practical scenario targeted in this work. To further address the risks in adversarial scenarios where metadata might be forged, we suggest that future work could consider cross-validating this method with image-content-based forensic features to enhance robustness. Second, this study does not explicitly model the correlations among EXIF metadata tags. A deeper investigation into such inter-tag relationships may further enhance the discriminative ability and robustness of metadata-based forensic analysis.

Finally, looking beyond specific limitations, we emphasize the potential for automated feature discovery in the era of complex forensic data structures. Recent interdisciplinary research [39] has highlighted the synergy between Large Language Models (LLMs) and Evolutionary Algorithms (EAs) as a robust path for feature engineering. Specifically, LLM-enhanced evolutionary frameworks could enable the automated interpretation of semantic nuances in heterogeneous metadata tags while leveraging evolutionary search capabilities to identify optimal feature combinations without manual intervention. Exploring such adaptive frameworks represents a promising direction for extending the proposed method to handle the growing complexity and scale of future multimedia forensic tasks.

Furthermore, expanding the verification dimension from the purely digital to the physical domain represents another vital frontier. As highlighted in the recent review by Shi and Shen [40], advanced haptic sensing technologies enable the precise capture of physical user–device interactions. Inspired by these mechanisms, we suggest that future forensic frameworks could evolve into multi-modal systems that correlate digital metadata with physical sensor logs, such as haptic signals or tactile interaction signatures recorded during the capture process. This “physically grounded” approach would significantly enhance robustness by verifying the actual physical presence of the device during image acquisition, thereby ensuring the integrity of the identified source.

## Figures and Tables

**Table 1:** Examples of irrelevant metadata.

Categories	Description	Example Attributes
Timestamp and geolocation information	It records the timestamp of the image capture and the geographic location.	CreateDate, DateCreated, DateTimeOriginal, GPSAltitude, GPSDateTime
Device information	It includes information about the device manufacturer, model, and software version	DeviceManufacturer, DeviceModel, ExifToolVersion, LensMake, Make
Image size information	It describes information related to the image’s size, resolution, and thumbnail	ImageHeight, ImageSize, ImageWidth, ThumbnailImage, ThumbnailLength, ThumbnailOffset

**Table 2:** Examples of the retained metadata.

Categories	Description	Example Attributes
Exposure parameter information	These parameters determine the brightness and sharpness of the image	ISO, ShutterSpeed, Aperture, LightValue, ExposureTime
Optical parameter information	It represents parameters related to the optical characteristics and physical state of the camera lens	FocalLength, FocalLength35efl, FocalLengthIn35mmFormat, HyperfocalDistance
Internal camera processing parameters information	It represents the parameters generated by the camera’s internal image processing algorithms and settings	WhiteBalance, MeteringMode, SceneCaptureType, SceneType

**Table 3:** Comparison of source camera identification performance at brand and model levels. All values are reported in percent (%). The equivalence class method identifies source cameras by analyzing EXIF metadata features, with its evaluation metric being the uniqueness ratio of device configurations, while the proposed method is evaluated by average accuracy.

Level	Method	ForensiCam-215K [10]	VISION [33]	HDRPlus [35]
Brand	Equivalence Class [36]	84.7	100	93.8
	Proposed	99.74	100	99.98
Model	Equivalence Class [36]	55	73.8	84.4
	Proposed	96.4	98.31	99.97

**Table 4:** Performance comparison with a deep learning baseline (BSA+CSA) on brand-level and model-level identification accuracy across three datasets. All values are reported in percent (%).

Dataset	Task	Deep Learning (BSA+CSA)	Proposed Method (Decision Tree)
VISION	Brand	99.85	100
	Model	98.1	98.31
HDRPlus	Brand	100	99.98
	Model	100	99.97
ForensiCam-215K	Brand	99.75	99.74
	Model	95.73	96.4

**Table 5:** Key metadata analysis for PRNU stability based on a decision tree model.

Metadata	Role in Decision Tree	Key Findings
LightValue	Root split node	When the value is less than or equal to 8.45, the sample is classified as a FLAT image. Otherwise, it proceeds to the subsequent node for further segmentation
ImageDescription	Key splitting node	The “sdr” is associated with low PCE anomalous samples, the “dav” consistently corresponds to high PCE normal samples, the “dsr” exhibits mixed characteristics
Compression	Part of complete decision process	In conjunction with other features
ExposureTime	Part of complete decision process	In conjunction with other features
ShutterSpeed	Part of complete decision process	In conjunction with other features

## Data Availability

The original data presented in the study are openly available in: The ForensiCam-215K dataset at [10], in VISION dataset at [33], and in HDRPlus dataset at https://github.com/JJL-a/HDRPlus (accessed on 13 January 2026).
